# HSP90 inhibition overcomes *EGFR* amplification‐induced resistance to third‐generation EGFR‐TKIs

**DOI:** 10.1111/1759-7714.13839

**Published:** 2021-01-20

**Authors:** Sho Watanabe, Yasushi Goto, Hiroyuki Yasuda, Takashi Kohno, Noriko Motoi, Yuichiro Ohe, Hiroyoshi Nishikawa, Susumu S. Kobayashi, Kazuyoshi Kuwano, Yosuke Togashi

**Affiliations:** ^1^ Division of Cancer Immunology Exploratory Oncology Research & Clinical Trial Center (EPOC), National Cancer Center Chiba Japan; ^2^ Department of Thoracic Oncology National Cancer Center Hospital Tokyo Japan; ^3^ Department of Respiratory Medicine Jikei University of Medicine Tokyo Japan; ^4^ Division of Pulmonary Medicine, Department of Medicine Keio University, School of Medicine Tokyo Japan; ^5^ Genome Biology Exploratory Oncology Research & Clinical Trial Center (EPOC), National Cancer Center Chiba Japan; ^6^ Pathology and Clinical Laboratories National Cancer Center Hospital Tokyo Japan; ^7^ Translational Genomics, Research Institute Exploratory Oncology Research & Clinical Trial Center (EPOC), National Cancer Center Chiba Japan; ^8^ Department of Medicine Beth Israel Deaconess Medical Center Boston Massachusetts USA

**Keywords:** acquired resistance, and heat shock protein 90, epidermal growth factor receptor, epidermal growth factor receptor amplification, epidermal growth factor receptor‐tyrosine kinase inhibitor

## Abstract

**Background:**

Patients with non‐small cell lung cancer (NSCLC) harboring activating *EGFR* mutations are sensitive to epidermal growth factor receptor‐tyrosine kinase inhibitors (EGFR‐TKIs) but inevitably develop resistance to the inhibitors mostly through acquisition of the secondary *T790M* mutation. Although third‐generation EGFR‐TKIs overcome this resistance by selectively inhibiting EGFR with EGFR‐TKI‐sensitizing and *T790M* mutations, acquired resistance to third‐generation EGFR‐TKIs invariably develops.

**Methods:**

Next‐generation sequencing (NGS) and fluorescence in situ hybridization (FISH) analysis were performed in an *EGFR T790M*‐mutated NSCLC patient who had progressed after a third‐generation EGFR‐TKI, TAS‐121. *EGFR*‐mutated cell lines were subjected to a cell proliferation assay and western blotting analysis with EGFR‐TKIs and a heat shock protein 90 (HSP90) inhibitor.

**Results:**

NGS and FISH analysis revealed *EGFR* amplification in the resistant cancer cells. While *EGFR L858R/T90M*‐mutated cell line was sensitive to osimertinib or TAS‐121 in vitro, *EGFR*‐overexpressing cell lines displayed resistance to these EGFR‐TKIs. Western blot analysis showed that EGFR phosphorylation and overexpression of *EGFR* in cell lines was not suppressed by third‐generation EGFR‐TKIs. In contrast, an HSP90 inhibitor reduced total and phosphorylated EGFR and inhibited the proliferation of resistant cell lines.

**Conclusions:**

*EGFR* amplification confers resistance to third‐generation EGFR‐TKIs which can be overcome by HSP90 inhibition. The results provide a preclinical rationale for the use of HSP90 inhibitors to overcome *EGFR* amplification‐mediated resistance.

## Graphical abstract

Third‐generation EGFR‐TKIs overcome EGFR T790M‐meditated resistance. However, amplified EGFR confers resistance to third‐generation EGFR‐TKIs. Inhibiting HSP90, a chaperon for EGFR, suppresses the proliferation of EGFR‐amplified cells by degrading EGFR in vitro, providing a preclinical rationale for the use of HSP90 inhibitors to overcome the EGFR amplification‐meditated resistance.

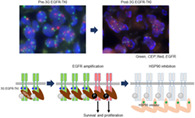

## INTRODUCTION

Epidermal growth factor receptor (*EGFR*) mutations are commonly found in non‐small cell lung cancer (NSCLC), with a prevalence of 10%–20% in Caucasian patients and 30%–40% in Asian patients with advanced NSCLC.^1^, ^2^ Upon mutation of the tyrosine kinase domains of *EGFR*, EGFR undergoes conformational changes, and its equilibrium is shifted towards a ligand‐independent activated state,^3^ which results in cell proliferation or survival.^4^ Activating *EGFR* mutations (in‐frame deletions in exon 19 and a point mutation in exon 21) trigger tumorigenesis and are a major determinant of susceptibility to EGFR tyrosine kinase inhibitors (EGFR‐TKIs).^5^ Numerous clinical trials have shown the superior efficacy of first‐generation EGFR‐TKIs (gefitinib and erlotinib) or second‐generation EGFR‐TKIs (afatinib) compared with chemotherapy^6^, ^7^, ^8^, ^9^, ^10^, ^11^ and have established these agents as the standard of care for advanced *EGFR*‐mutated NSCLC.

Despite their marked response to EGFR‐TKIs, *EGFR*‐mutated NSCLCs inevitably develop resistance to these inhibitors after approximately 8–13 months of treatment.^5^ Among the resistance mechanisms, the *EGFR* T790M mutation is predominant, occurring in approximately half of *EGFR*‐mutated NSCLC cases.^5^, ^12^, ^13^ However, this limitation has been overcome by the introduction of third‐generation EGFR‐TKIs, such as osimertinib. These compounds covalently bind to the C797 residue within the mutant EGFR kinase domain, irreversibly binding to the ATP‐binding site while sparing wild‐type EGFR.^14^, ^15^, ^16^ These characteristics of third‐generation EGFR‐TKIs have led to their significant efficacy and decreased toxicity in clinical trials,^17^, ^18^ and osimertinib has been approved for the treatment of *EGFR*‐positive NSCLC patients.^19^

Similar to patients treated with earlier‐generation EGFR‐TKIs, those receiving third‐generation EGFR‐TKIs invariably develop drug resistance. Identification of the resistance mechanisms is crucial for improving outcomes in patients with *EGFR*‐mutated NSCLC. Heterogeneous mechanisms underlying resistance to third‐generation EGFR‐TKIs have been reported; these include the tertiary *EGFR* C797S mutation; amplification of *MET* or *HER2*; mutations in *PIK3CA*, *ALK*, or *BRAF*; and *RET* fusions.^20^ However, the resistance mechanisms are not fully understood and are unknown in 30–50% of cases,^21^ necessitating their further investigation.

Heat shock proteins (HSPs) assist in the folding of nascent polypeptides into a functional conformation, thus facilitating protein stability and turnover, which are necessary for the intracellular localization and function of proteins.^22^ The HSP90 chaperone machinery, a key regulator of proteostasis, impairs apoptotic signaling in cancer cells.^23^ Since EGFR is a client protein for the HSP90 chaperone, a strategy of targeting HSP90 has been evaluated in *EGFR*‐mutated NSCLC.^24^ While clinical trials have shown the activity of HSP90 inhibitors in NSCLC patients harboring *EGFR* mutations,^25^, ^26^ whether inhibition of HSP90 can overcome acquired resistance to third‐generation EGFR‐TKIs remains to be determined.

Here, we report a resistance mechanism of *EGFR* amplification in a patient with *EGFR* T790M‐mutated NSCLC who developed acquired resistance to a third‐generation EGFR‐TKI, TAS‐121.^16^ We then investigated the role of *EGFR* amplification in this resistance in vitro. Furthermore, we evaluated the sensitivity of this tumor to an HSP90 inhibitor (TAS‐116)^27^ to evaluate the therapeutic possibility of a potent HSP90 inhibitor.

## METHODS

### Patient and samples

Tumor samples were obtained by biopsy and autopsy from a patient with metastatic lung adenocarcinoma harboring an *EGFR* L858R mutation and were analyzed by next‐generation sequencing (NGS) and/or fluorescence in situ hybridization (FISH).

### NGS of clinical samples

NGS (NCC OncoPanel v3, Agilent Technologies) was performed using formalin‐fixed, paraffin‐embedded (FFPE) samples obtained from a progressive liver lesion during TAS‐121 therapy as previously described.^28^, ^29^ Sequenced genes are summarized in Table S1.

### FISH analysis

We conducted FISH analysis for *EGFR* with a Vysis EGFR Dual Color Probe‐Hyb Set (Abbott Laboratories) using FFPE samples from pre‐TAS‐121‐treatment liver lesions, post‐TAS‐121‐treatment liver lesions, and autopsied lung and liver lesions. *EGFR* amplification was indicated if the EGFR/CEP signal ratio was >2.0.^30^

### Cell lines and reagents

The HCC827 and H1975 cell lines (human NSCLC cell lines) were obtained from the American Type Culture Collection (ATCC), and the PC‐9 cell line (a human NSCLC cell line) was obtained from the European Collection of Authenticated Cell Cultures (ECACC). The PC9‐COR cell line was established from the PC‐9 cell line as previously reported.^31^ All cell lines were authenticated using the short tandem repeat method and were maintained in RPMI medium (FUJIFILM Wako Pure Chemical Corporation) supplemented with 10% fetal bovine serum (FBS; Cytiva, Marlborough, MA). The *EGFR* status of the cell lines is summarized in Table S2. All cell lines were used after they were confirmed to be negative for *Mycoplasma* contamination with a PCR Mycoplasma Detection Kit (TaKaRa Bio) according to the manufacturer's instructions. Erlotinib and osimertinib were obtained from Cayman Chemical Company. TAS‐121 and TAS‐116 were kindly provided by Taiho Pharmaceutical Co., Ltd.

### Cell proliferation assay

Cells were plated in 96‐well plates at a density of 2 × 10^3^ cells/well and incubated for 24 h. Cell proliferation was evaluated with a WST‐1 assay (TaKaRa Bio) after 72 h of treatment. The absorption of WST‐1 was measured at a wavelength of 450 nm with a reference wavelength of 690 nm in a microplate reader. Cell viability was calculated as the ratio of the absorbance value of the treated cells to that of the control cells and expressed as a percentage. Experiments were performed independently in triplicate.

### Establishment of the *EGFR* L858R/T790M‐overexpressing H1975 cell line

H1975 cells with overexpression of *EGFR* L858R/T790M (H1975‐LR/TM) were generated by retroviral transduction. In brief, packaging cells were transfected with pBABE‐puro‐*EGFR* L858R/T790M or a control pBABE‐puro‐mock vector (Addgene) and a VSV‐G vector (TaKaRa Bio) using Lipofectamine 3000 (Thermo Fisher Scientific). Viral supernatant was collected two days after transfection, and viral particles were transduced into H1975 cells.

### Western blotting

Subconfluent cells were washed with PBS and harvested with M‐PER (Thermo Fisher). Whole cell lysates were separated by SDS‐PAGE and transferred to a polyvinylidene fluoride membrane. After blocking, the membrane was probed with a primary antibody. After two rinses with TBS‐T buffer, the membrane was incubated with a horseradish peroxidase‐conjugated secondary antibody and washed. Immunoreactions were visualized using an ECL detection system and a LAS‐4000 (GE Healthcare). Antibodies are summarized in Table S3. Experiments were performed independently in at least triplicate.

### Apoptosis

Apoptosis was assessed using flowcytometry with FITC‐annexin V and 7‐AAD (Thermo Fisher Scientific). The staining reagents were diluted in accordance with the manufacturer's instructions. The stained cells were analyzed with the LSR Fortessa (BD Biosciences) and FlowJo software (BD Biosciences).

### Xenograft studies

Female BALB/c nu/nu nude mice (6–8 weeks old) were purchased from CLEA Japan. H1975 cells, H1975‐LR/TM, and PC9‐COR cells (1 × 10^6^) in 100 μl of RPMI with 100 μl of Matrigel were injected subcutaneously into the backs of mice, and the tumor volume was assessed twice a week using the formula “length × width^2^ × 0.5.” The mice were grouped when the tumor volume reached approximately 200–500 mm.^3^ TAS‐121 (12.5 mg/kg/day) and the vehicle were orally administered daily, and TAS‐116 (14 mg/kg/day) was orally administered five times a week thereafter. All the mice were maintained under specific pathogen‐free conditions in the animal facility of the Institute of Biophysics. The mouse experiments were approved by the Animal Committee for Animal Experimentation of the National Cancer Center. All the experiments met the guidelines of the US Public Health Service Policy on Humane Care and Use of Laboratory Animals.

### Statistical analysis

Continuous variables were analyzed using a *t*‐test. The relationship between the tumor volume curves were compared using a two‐way ANOVA. Statistical analyses were two‐tailed and performed with the Prism version 7 software (GraphPad Software, Inc., La Jolla, CA, USA). A *p*‐value of less than 0.05 was considered statistically significant.

## RESULTS

### Clinical course

**FIGURE 1:**
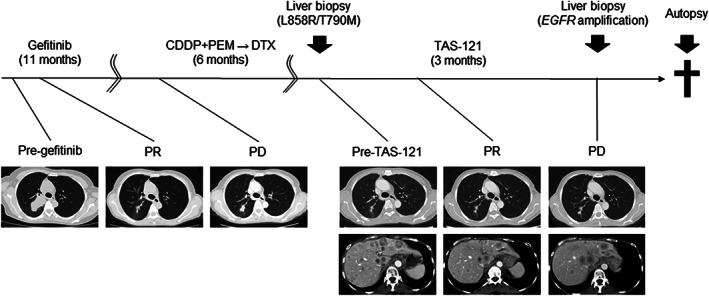
Clinical course of a 68‐year‐old woman with metastatic lung adenocarcinoma (cT4N2M1b) harboring an *EGFR* L858R mutation who received gefitinib and achieved a partial response (PR). Eleven months later, she experienced progressive disease (PD) and received chemotherapy. Liver biopsy of progressing liver metastases indicated a secondary mutation of *EGFR* T790M in addition to the primary mutation. She was treated with TAS‐121 in a phase I trial. Despite the initial response, PD was confirmed by CT which showed regrowth of liver lesions. She died four months after initiation of TAS‐121 treatment

A 68‐year‐old woman with metastatic lung adenocarcinoma (cT4N2M1b, stage IV) harboring the *EGFR* L858R mutation received gefitinib treatment and achieved a partial response (PR) (Figure 1). She experienced progressive disease (PD) 11 months later and subsequently received four cycles of chemotherapy with cisplatin and pemetrexed followed by pemetrexed maintenance therapy. Computed tomography (CT) after six months of chemotherapy showed disease progression with liver metastases. Tumor progression was also noted after completion of one cycle of docetaxel, and rebiopsy of a progressing liver lesion was performed. Tumor genotyping with the peptide nucleic acid‐locked nucleic acid (PNA‐LNA) PCR clamp method revealed a secondary mutation of *EGFR* T790M in addition to the primary *EGFR* L858R mutation. She was enrolled in a phase I trial of TAS‐121 (12 mg/day).^32^ Despite initial PR, PD was confirmed by CT after three months, indicating progression of liver metastases whereas lung lesions were stable. Rebiopsy of the progressive liver lesion was subsequently performed. She discontinued TAS‐121 and received best supportive care. She died four months after initiation of TAS‐121 treatment, and an autopsy was performed.

### *EGFR* amplified in patient with acquired resistance to TAS‐121

**FIGURE 2:**
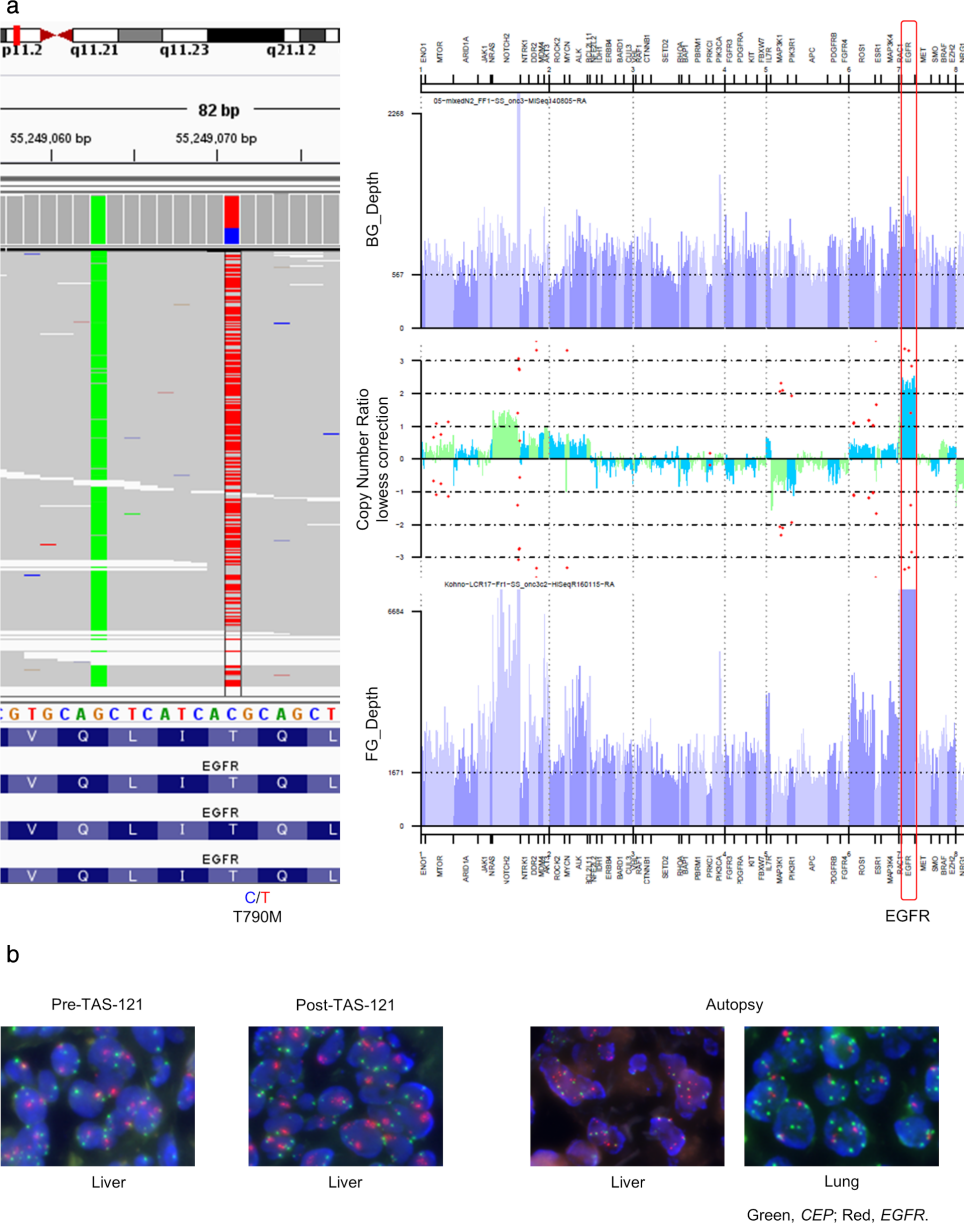
*EGFR* gene status. (a) Next‐generation sequencing (NGS) of the EGFR gene. The post‐TAS‐121‐treatment liver sample was analyzed using NGS. The *EGFR* mutation (left) and copy number gain (right, red) are shown. Blue, wild‐type allele; red, T790M‐mutated allele. (b) Fluorescence in situ hybridization (FISH). We conducted FISH analyses of *EGFR* using formalin‐fixed, paraffin‐embedded (FFPE) samples from the pre‐TAS‐121‐treatment liver lesion, the post‐TAS‐121‐treatment liver lesion, and autopsied lung and liver lesions. Green, *CEP*; red, *EGFR*

NGS of the progressive liver lesion post TAS‐121 treatment revealed *EGFR* amplification (log2 ratio = 2.2). Whereas both the wild‐type and T790M‐mutated *EGFR* alleles were amplified, the mutated allele was dominant (Figure 2(a)). In contrast, we were unable to find any typical genetic alterations that can cause resistance to third‐generation EGFR‐TKIs (i.e., *MET* amplification or *EGFR* C797S mutation; Tables S1 and S4). According to the definition of *EGFR* amplification, which is an EGFR/CEP signal ratio > 2.0 by FISH analysis,^30^ no *EGFR* amplification was identified in the pre‐TAS‐121‐treatment liver lesion (EGFR/CEP signal ratio, 1.7) but was detected in both the rebiopsied post‐TAS‐121‐treatment and autopsied liver lesions (2.1 and 3.0, respectively; Figure 2(b)). *EGFR* amplification was not observed in the autopsied lung lesions (1.0), which did not progress during TAS‐121 treatment. These findings suggest that the patient acquired resistance to TAS‐121 owing to the *EGFR* amplification mainly of the mutated allele.

### Third‐generation EGFR‐TKIs, including TAS‐121, effectively inhibit the proliferation of *EGFR*‐mutated NSCLC cells

**FIGURE 3:**
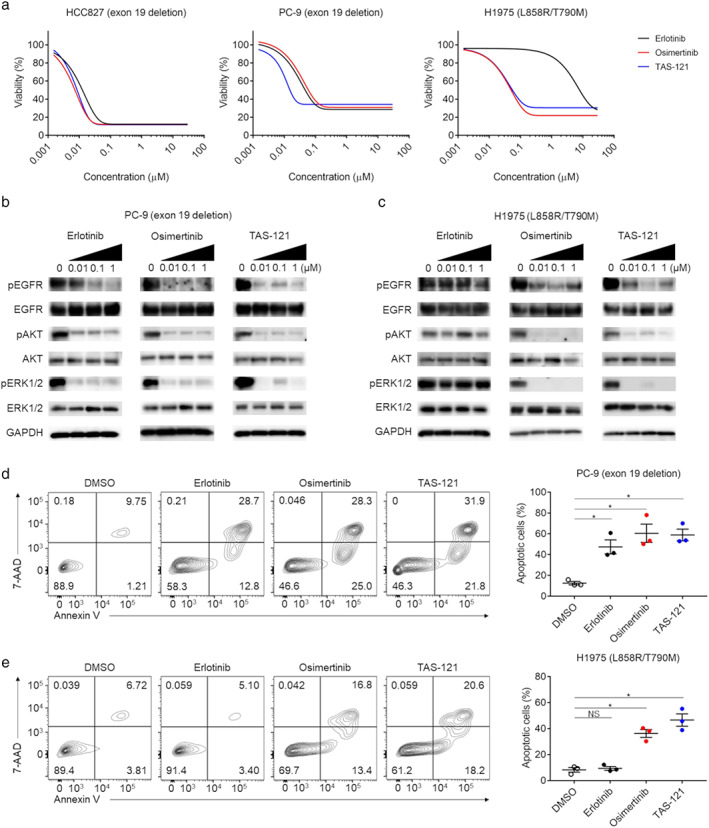
Sensitivities of *EGFR*‐mutated cell lines to EGFR‐TKIs. (a) Cell proliferation assay of several *EGFR*‐mutated cell lines under EGFR‐TKIs. HCC827 (left), PC‐9 (middle), and H1975 (right) cells were plated in 96‐well plates at a density of 2 × 10^3^ cells/well, and cell proliferation was evaluated with the WST‐1 assay following 72 h of treatment. Experiments were independently performed in triplicate, and the means are shown. Black, erlotinib; red, osimertinib; blue, TAS‐121. (b) and (c) Western blotting in PC‐9 (b) and H1975 cell lines (c). Cells were cultured with the indicated concentrations of EGFR‐TKIs for 3 h, and the cell lysates were then subjected to western blot analysis. GAPDH was used as the internal control. Representative data from three independent experiments are shown. (d) and (e), Apoptosis in the PC‐9 (d) and H1975 (e) cell lines. Apoptosis was assessed using flowcytometry with FITC‐annexin V and 7‐AAD after 72 h of treatment. Representative data from three independent experiments (left), and the mean and SEM values (right) are shown. *, *p* < 0.05; NS, not significant

To verify the efficacy of EGFR‐TKIs in *EGFR*‐mutated cell lines, we evaluated sensitivity to the inhibitors in vitro. As expected, the HCC827 and PC‐9 cell lines carrying the activating *EGFR* exon 19 deletion mutation were sensitive to erlotinib (a first‐generation EGFR‐TKI) and to osimertinib and TAS‐121 (third‐generation EGFR‐TKIs) (Figure 3(a)). The proliferation of H1975 cells harboring the T790M resistance and L858R sensitizing mutations was inhibited by osimertinib and TAS‐121 but not by erlotinib (Figure 3(a)). Consistent with this result, western blot analysis showed that osimertinib and TAS‐121 reduced phosphorylation of EGFR and the downstream AKT and ERK in the H1975 cell line in a dose‐dependent manner as well as in the PC‐9 cell line, whereas erlotinib did not decrease phosphorylation in the H1975 cell line (Figure 3(b) and (c)). Apoptosis was significantly induced by all EGFR‐TKIs in the PC‐9 cell line (Figure 3(d)). In contrast, erlotinib did not induce apoptosis, whereas TAS‐121 as well as osimertinib significantly induced apoptosis in the H1975 cell line (Figure 3(e)). These findings confirm that TAS‐121, a third‐generation EGFR‐TKI, overcomes the *EGFR* T790M‐driven resistance to erlotinib by a mechanism similar to that of osimertinib.^16^

### *EGFR* amplification meditates resistance to third‐generation EGFR‐TKIs

**FIGURE 4:**
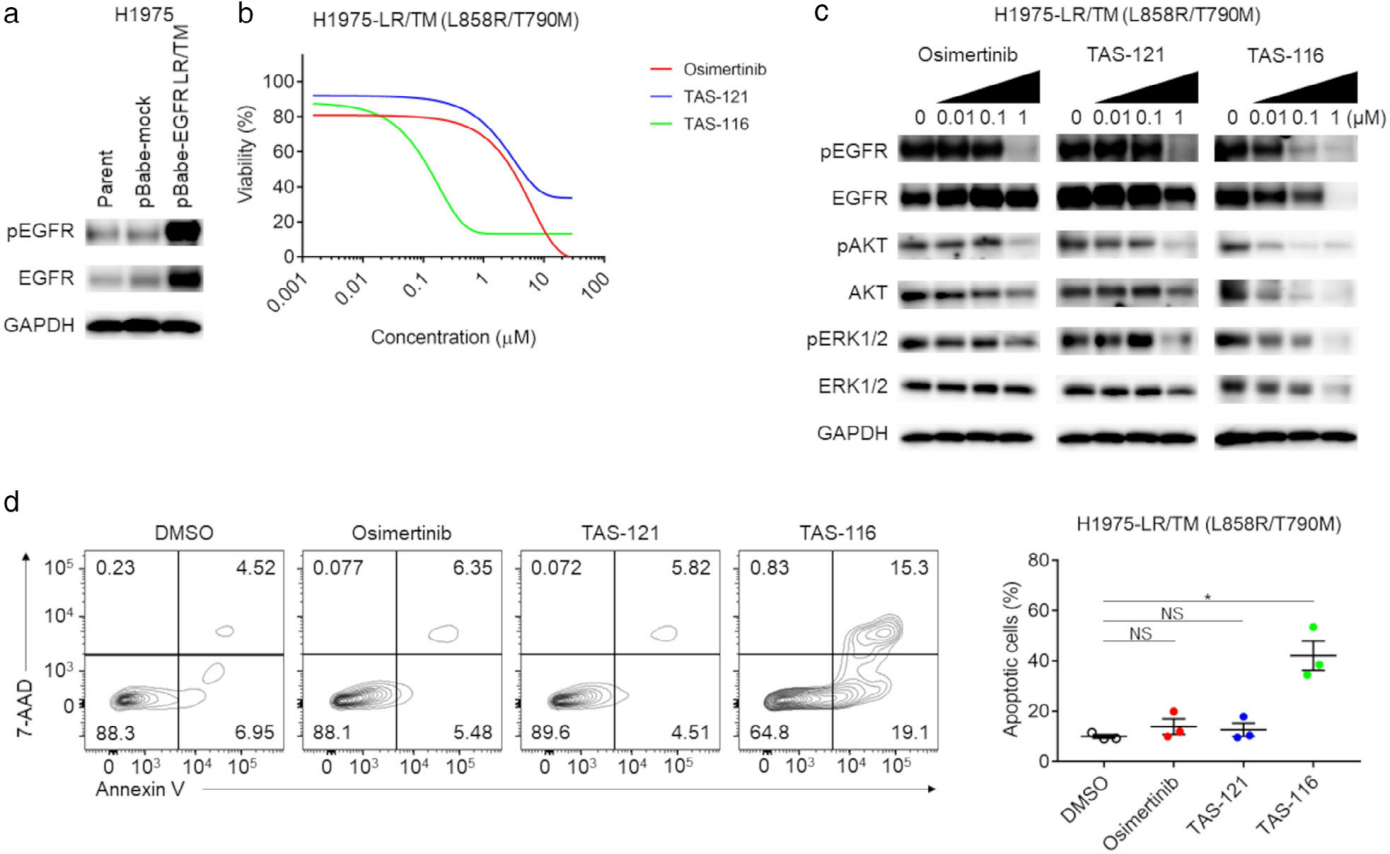
Sensitivities of the H1975‐LR/TM cell line to EGFR‐TKIs or TAS‐116. (a) Western blotting in H1975 cell lines. H1975 cells with overexpression of *EGFR* L858R/T790M (H1975‐LR/TM) were generated by retroviral transduction, and EGFR expression was analyzed by western blotting. GAPDH was used as the internal control. Representative data from three independent experiments are shown. (b) Cell proliferation assay of the H1975‐LR/TM cell line under EGFR‐TKIs or TAS‐116. Cells were plated in 96‐well plates at a density of 2 × 10^3^ cells/well, and cell proliferation was evaluated with a WST‐1 assay after 72 h of treatment. Experiments were independently performed in triplicate, and the means are shown. Red, osimertinib; blue, TAS‐121; green, TAS‐116. (c) Western blot analysis of the H1975‐LR/TM cell line. Cells were cultured with the indicated concentrations of EGFR‐TKIs for 3 h or TAS‐116 for 48 h, and the cell lysates were then subjected to western blot analysis. GAPDH was used as the internal control. Representative data from three independent experiments are shown. (d) Apoptosis in the H1975 cell‐LR/TM cell line. Apoptosis was assessed using flow cytometry with FITC‐annexin V and 7‐AAD after 72 h of treatment. Representative data from three independent experiments (left), and the mean and SEM values (right) are shown. *, *p* < 0.05; NS, not significant

The results of NGS data analysis and FISH analysis in our patient suggested that amplification of *EGFR*, especially the T790M mutant, occurred upon resistance to TAS‐121. Thus, we used retroviral transduction to generate an H1975 cell line with overexpression of L858R/T790M‐mutated *EGFR* (H1975‐LR/TM), and evaluated inhibitor sensitivities in vitro. Figure 4(a) shows that H1975‐LR/TM cells contained higher levels of phosphorylated and total EGFR than control cells. In the cell proliferation assay, the H1975‐LR/TM cell line was resistant to both TAS‐121 and osimertinib (Figure 4(b)). These results were supported by those of western blot analysis; that is, TAS‐121 and osimertinib did not inhibit phosphorylation of EGFR and the downstream AKT and ERK in the H1975‐LR/TM cell line (Figure 4(c)). Consistently, apoptosis was not induced by TAS‐121 and osimertinib in this cell line (Figure 4(d)). These findings suggest that amplification of the mutated *EGFR* gene mediated the resistance to TAS‐121 in this patient.

**FIGURE 5:**
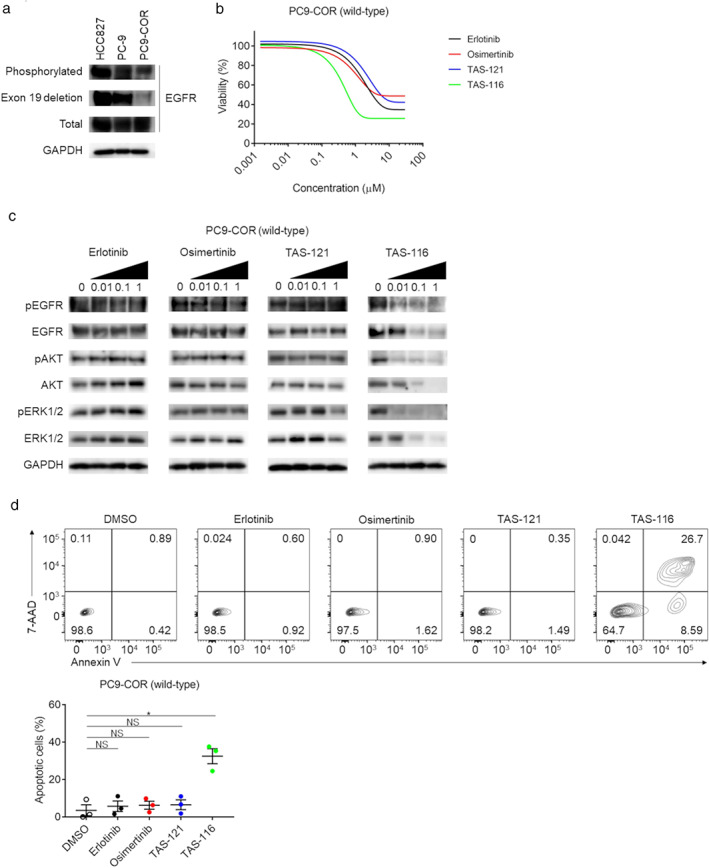
Sensitivities of the PC9‐COR cell line to EGFR‐TKIs and TAS‐116. (a) Western blot analysis of the cell lines. EGFR with exon 19 deletion was detected using a specific antibody. GAPDH was used as the internal control. Representative data from three independent experiments are shown. (b) Cell proliferation assay of the PC9‐COR cell line under treatment with EGFR‐TKIs or TAS‐116. Cells were plated in 96‐well plates at a density of 2 × 10^3^ cells/well, and cell proliferation was evaluated with a WST‐1 assay after 72 h of treatment. Experiments were performed independently in triplicate, and the means are shown. Black, erlotinib; red, osimertinib; blue, TAS‐121; green, TAS‐116. (c) Western blot analysis of the PC9‐COR cell line. Cells were cultured with the indicated concentrations of EGFR‐TKIs for 3 h or with TAS‐116 for 48 h, and the cell lysates were then subjected to western blot analysis. GAPDH was used as the internal control. Representative data from three independent experiments are shown. (d) Apoptosis in the PC9‐COR cell line. Apoptosis was assessed using flowcytometry with FITC‐annexin V and 7‐AAD after 72 h of treatment. Representative data from three independent experiments (top), and the mean and SEM values (bottom) are shown. *, *p* < 0.05; NS, not significant

The image generated by Interactive Genome Viewer showing the possible coemergence of the wild‐type *EGFR* allele prompted us to evaluate the effects of EGFR‐TKIs on the PC9‐COR cell line with amplification of wild‐type *EGFR*, which was previously established using rociletinib as a clone resistant to erlotinib and third‐generation EGFR‐TKIs^31^ (Figure 2(a)). We confirmed the expression of not mutated but wild‐type EGFR in PC9‐COR cells (Figure 5(a)). Accordingly, the PC9‐COR cell line displayed resistance to TAS‐121 as well as to erlotinib and osimertinib (Figure 5(b)), and western blot analysis showed that no EGFR‐TKI reduced phosphorylation of EGFR and the downstream AKT and ERK (Figure 5(c)). Apoptosis was not induced by these EGFR‐TKIs in this cell line (Figure 5(d)). These findings are consistent with those of a previous study showing that amplification of wild‐type *EGFR* also mediates resistance to third‐generation EGFR‐TKIs.^31^ Taken together, these results indicate that amplification of both mutated and wild‐type *EGFR* can mediate resistance to third‐generation EGFR‐TKIs.

### HSP90 inhibitor, TAS‐116, overcomes *EGFR* amplification‐mediated acquired resistance to third‐generation EGFR‐TKIs

**FIGURE 6:**
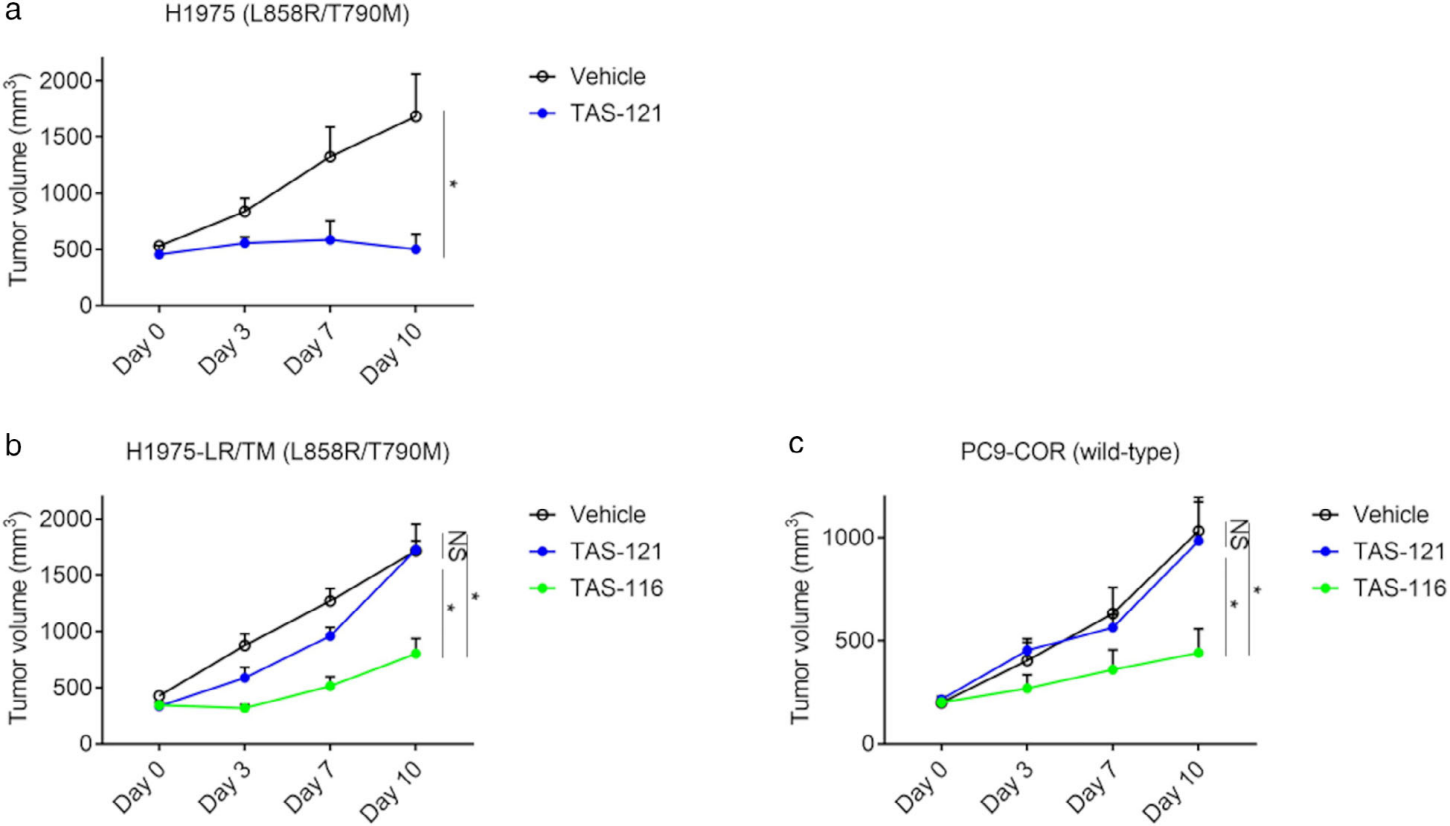
In vivo effects of TAS‐116 against H1975‐LR/TM and PC9‐COR tumors. (a) in vivo efficacy of TAS‐121 against H1975 tumors. Cells (1 × 10^6^) in 100 μl of RPMI with 100 μl of Matrigel were injected subcutaneously, and the tumor volume was assessed twice a week using the formula “length × width^2^ × 0.5.” The mice were grouped when the tumor volume reached approximately 200–500 mm^3^. TAS‐121 (12.5 mg/kg/day) and the vehicle were orally administered daily thereafter. (b) and (c) in vivo efficacy of TAS‐116 against H1975‐LR/TM (b) and PC9‐COR (c) tumors. in vivo experiments were performed as described in A. TAS‐116 (14 mg/kg/day) was administered orally five times a week. The mean and SEM values are shown. *, *p* < 0.05; NS, not significant

To explore the possibility that HSP90 inhibition can overcome *EGFR* amplification‐mediated resistance, we analyzed the sensitivities of PC‐9, H1975, H1975‐LR/TM and PC9‐COR cell lines to the HSP90 inhibitor TAS‐116 in vitro. Parent cell lines (PC‐9 and H1975) were sensitive to TAS‐116 (Figure S1). Additionally, the viability of both H1975‐LR/TM and PC9‐COR cells was compromised by treatment with the HSP90 inhibitor (Figure 4(b) and Figure 5(b)). Importantly, TAS‐116 was highly active against these cell lines at therapeutic concentrations of 1–3 μM.^33^, ^34^ In contrast, the combination efficacy of TAS‐121 and TAS‐116 was not observed in both the H1975 and H1975‐LR/TM cell lines (Figure S1). Consistent with this finding, TAS‐116 greatly reduced the levels of both total and phosphorylated EGFR and the downstream AKT and ERK in these cell lines in a concentration‐dependent manner (Figure 4(c) and Figure 5(c)). Apoptosis was also significantly induced by TAS‐116 (Figures 4(d) and 5(d)). Furthermore, the in vivo effects of TAS‐116 against both H1975‐LR/TM and PC9‐COR tumors that were resistant to TAS‐121 were also observed (Figure 6(a)–(c)). These results provide a preclinical rationale for the use of HSP90 inhibitors to overcome the acquired resistance to third‐generation EGFR‐TKIs induced by *EGFR* amplification.

## DISCUSSION

In this study, we identified mutated *EGFR* amplification as a resistance mechanism in a patient with *EGFR* T790M‐positive NSCLC who experienced PD on a third‐generation EGFR‐TKI, TAS‐121. In vitro, amplification of not only wild‐type but also mutated *EGFR* induced resistance. This is the first case of mutated *EGFR* amplification‐mediated resistance in a patient treated with TAS‐121, although this mechanism has been reported in patients treated with other third‐generation EGFR‐TKIs.^35^, ^36^ In addition, TAS‐116 overcame this resistance in vitro and in vivo, indicating that HSP90 inhibition is a therapeutic strategy for patients with *EGFR* amplification‐mediated resistance to third‐generation EGFR‐TKIs.

Acquired resistance has been a challenge in EGFR‐TKI therapy. Initial characterization of resistance to earlier‐generation EGFR‐TKIs identified *EGFR* T790M mutation as a predominant resistance mechanism and led to the development of third‐generation EGFR‐TKIs.^5^ However, the efficacy of third‐generation EGFR‐TKIs has been limited by the occurrence of secondary resistance.^20^ Numerous studies have shown the heterogeneity of mechanisms underlying resistance to third‐generation EGFR‐TKIs. In addition to the tertiary resistance *EGFR* C797S mutation, somatic copy number alterations account for a substantial portion of resistance.^21^, ^37^ From our NGS data, we identified *EGFR* amplification as a resistance mechanism, but no other alterations, including *MET* amplification and *EGFR* C797S mutation, were identified as resistance mechanisms. Consistent with our findings, a prior study reported that amplification of mutated *EGFR* emerged in 39% of plasma samples from patients with acquired resistance to earlier‐generation EGFR‐TKIs and 9% of those from patients with resistance to a third‐generation EGFR‐TKI.^38^ Amplification of the T790M allele was also confirmed in 23% of biopsy samples from patients with T790M‐positive NSCLC at development of resistance to a third‐generation EGFR‐TKI.^35^ Understandably, amplification of wild‐type *EGFR* can confer resistance to third‐generation EGFR‐TKIs because third‐generation EGFR‐TKIs selectively inhibit mutated EGFR and allow wild‐type EGFR to escape inhibition.^5^ Indeed, amplification of wild‐type *EGFR* was reported to decrease the sensitivity of *EGFR*‐mutated cancer cells to third‐generation EGFR‐TKIs in vitro^31^, ^38^, ^39^ and to cause resistance to osimertinib in a patient with T790M‐positive NSCLC.^40^ Conversely, in the clinical setting, accumulating evidence, including our case, indicates that amplification of mutated *EGFR* drives resistance to these inhibitors despite the potent inhibition of mutant EGFR by third‐generation EGFR‐TKIs.^35^, ^38^, ^41^ These conflicting results highlight the need for an improved understanding of *EGFR* amplification‐mediated resistance. Here, we found that the overexpression of mutated *EGFR* outpaced the inhibitory activity of third‐generation EGFR‐TKIs, providing additional insight into the interplay between *EGFR* amplification and the response to EGFR‐TKIs. In addition, the increasing use of circulating tumor DNA in blood to clarify resistance mechanisms may favor the reporting of amplification of mutated *EGFR*, because the inevitable contamination by nonmalignant cells hinders clinicians from accurately analyzing the copy numbers of wild‐type genes.^31^ Thus, the association between the copy numbers of mutated *EGFR* and EGFR inhibition needs further investigation to determine minimum amplification threshold for induction of resistance, and analyzing not only *EGFR* mutations but also *EGFR* copy numbers can facilitate access to optimal therapies in clinical settings.

In our patient, CT on the failure of TAS‐121 therapy showed mixed response to the agent; among the multiple lesions in the lung and liver, only liver metastasis progressed, and the lung lesions did not change in size. In the FISH analysis, *EGFR* amplification was found in the resistant liver lesions but not in the lung lesions, which suggests that heterogeneous cancer cells emerged with resistant *EGFR*‐amplified cancer cells in the liver metastases. Indeed, the results of an ongoing clinical trial raised the possibility that a focal copy number gain occurred subclonally upon the development of osimertinib resistance and was spatially and temporally separated from common resistance mechanisms, such as C797S mutation.^42^ Thus, the *EGFR* amplification in resistant liver lesions in our patient could reflect the evolutionary process of subclones selected by the potent EGFR‐inhibitory effects of TAS‐121 and demanded to selectively target *EGFR* amplification to overcome the resistance.

HSP90 inhibitors have exhibited potent antitumor activities in various preclinical models by destabilizing HSP90 client proteins.^43^ Importantly, mutated EGFR proteins are particularly reliant on the chaperone activity of HSP90 for their conformational stability and function,^44^ which led the H1975‐LR/TM cell line harboring the mutated *EGFR* amplification to have higher sensitivity than the PC9‐COR cell line with wild‐type *EGFR* amplification to TAS‐116 in our present study. In clinical trials, luminespib, a member of another class of HSP90 inhibitors, exhibited activity against *EGFR*‐mutated NSCLC.^25^, ^26^ Consistent with this finding, our present study shows that TAS‐116 exhibited efficacies against parent *EGFR*‐mutated cell lines sensitive to EGFR‐TKIs. In addition, HSP90 inhibition alone reportedly overcame the *MET* amplification‐ or HGF‐induced resistance to EGFR‐TKIs in vitro.^45^, ^46^ Luminespib combined with osimertinib exhibited a marked efficacy for intrinsic resistance to osimertinib by decreasing the phosphorylation of EGFR and MET and downregulating their downstream pathways.^47^ Our present study also showed that AKT and ERK, the downstream signals in the EGFR pathway, were degraded by HSP90 inhibition using TAS‐116, which supports the use of HSP90 inhibitors to overcome the resistance to EGFR‐TKIs.

Our patient received third‐generation EGFR‐TKI after acquired resistance to gefitinib because at that time no first‐line osimertinib therapy had been established. Consequently, we identified EGFR amplification as a resistance mechanism to fourth‐line third‐generation EGFR‐TKI. However, first‐line osimertinib therapy is a standard of care in the current clinical setting, and whether *EGFR* amplification can confer resistance to third‐generation EGFR‐TKIs regardless of the treatment lines with EGFR‐TKIs remains uncertain. In a previous study, *EGFR* amplification induced resistance to first‐line third‐generation EGFR‐TKIs.^21^ An osimertinib‐resistant PC9‐COR cell line represented the resistance to first‐line third‐generation EGFR‐TKIs,^31^ which was overcome by TAS‐116 in our study. Thus, HSP90 inhibitors can also overcome *EGFR* amplification‐mediated resistance to first‐line third‐generation EGFR‐TKIs.

HSP90 inhibitors have shown limited efficacy as single agents.^43^ Undesirable off‐target and/or HSP90‐related adverse events could account for this discrepancy via the need to limit the drug concentrations to levels insufficient to efficiently suppress intratumoral HSP90 activity.^24^ The most common adverse event in patients receiving HSP90 inhibitors was visual disorders due to sustained HSP90 inhibition in the retina.^48^ TAS‐116 possesses a distinct advantage over other HSP90 inhibitors, as its distribution in retinal tissue is lower than that in plasma, and it is rapidly eliminated from the retina.^27^ Indeed, eye disorders were not clinically significant in trials for patients with advanced solid tumors.^33^, ^34^ Therefore, further studies should focus on TAS‐116 as a promising therapeutic option for *EGFR*‐mutated lung cancer. In particular, patient‐derived experiments (xenograft model and patient‐derived cell line or organoid) would be an alternative method to validate their efficacy.

Another strategy to overcome *EGFR* amplification‐meditated resistance is the addition of cetuximab, a human–mouse chimeric antibody that binds to the extracellular domain of EGFR, to third‐generation EGFR‐TKIs. The efficacy of cetuximab combined with afatinib, a second‐generation EGFR‐TKI, has been reported in previous studies.^49^, ^50^, ^51^ By inhibiting EGF‐induced activation of wild‐type EGFR in PC9‐COR cell line^31^ or dimerization of mutated EGFR induced by EGFR‐TKI in erlotinib‐resistant *EGFR*‐mutated cell lines,^52^ cetuximab can enhance the inhibition of third‐generation EGFR‐TKIs. However, the addition of cetuximab to afatinib did not improve the outcomes in previously untreated *EGFR*‐mutant NSCLC patients but led to greater toxicity, including a 72% incidence rate of grade ≥ 3 treatment‐related adverse events.^53^

In summary, this study demonstrated that mutated *EGFR* amplification led to resistance to a third‐generation EGFR‐TKI, TAS‐121, in a patient with *EGFR* T790M‐positive NSCLC. Targeting the HSP90 chaperone using TAS‐116 overcame this resistance in vitro and in vivo, indicating a preclinical rationale for the use of HSP90 inhibitors in patients with *EGFR* amplification‐mediated resistance to third‐generation EGFR‐TKIs. Additional investigations into *EGFR* amplification‐mediated resistance and the efficacy and safety of HSP90 inhibition are warranted for the development of optimal therapies.

## CONFLICT OF INTEREST

Y.G. received research grant related to this work and honoraria outside this work from Taiho Pharmaceutical. H.N. and S.S.K. received research grant from Taiho Pharmaceutical outside this work. Y.T. received research grants and honoraria from Ono Pharmaceutical and Bristol‐Myers Squibb, research grants from Daiichi‐Sankyo and KOTAI Biotechnologies Inc, and honoraria from, AstraZeneca, Chugai Pharmaceutical, and MSD outside this work. All other authors have no competing financial interests.

## Supporting information


**Appendix S1** Supporting information
