# Supplementary material for: HSP90 inhibition overcomes EGFR amplification‐induced resistance to third‐generation EGFR‐TKIs
Source: Thorac Cancer. 2021 Jan 20;12(5):631–42. doi: 10.1111/1759-7714.13839 (PMC7919131; doi:10.1111/1759-7714.13839)
Supplement: Supplementary file 1 — Appendix S1 Supporting information [file TCA-12-631-s001.docx]

**Supplementary Figure S1.** Sensitivities of *EGFR*-mutated NSCLC cell lines to TAS-116 with or without TAS-121 (0.01 µM).


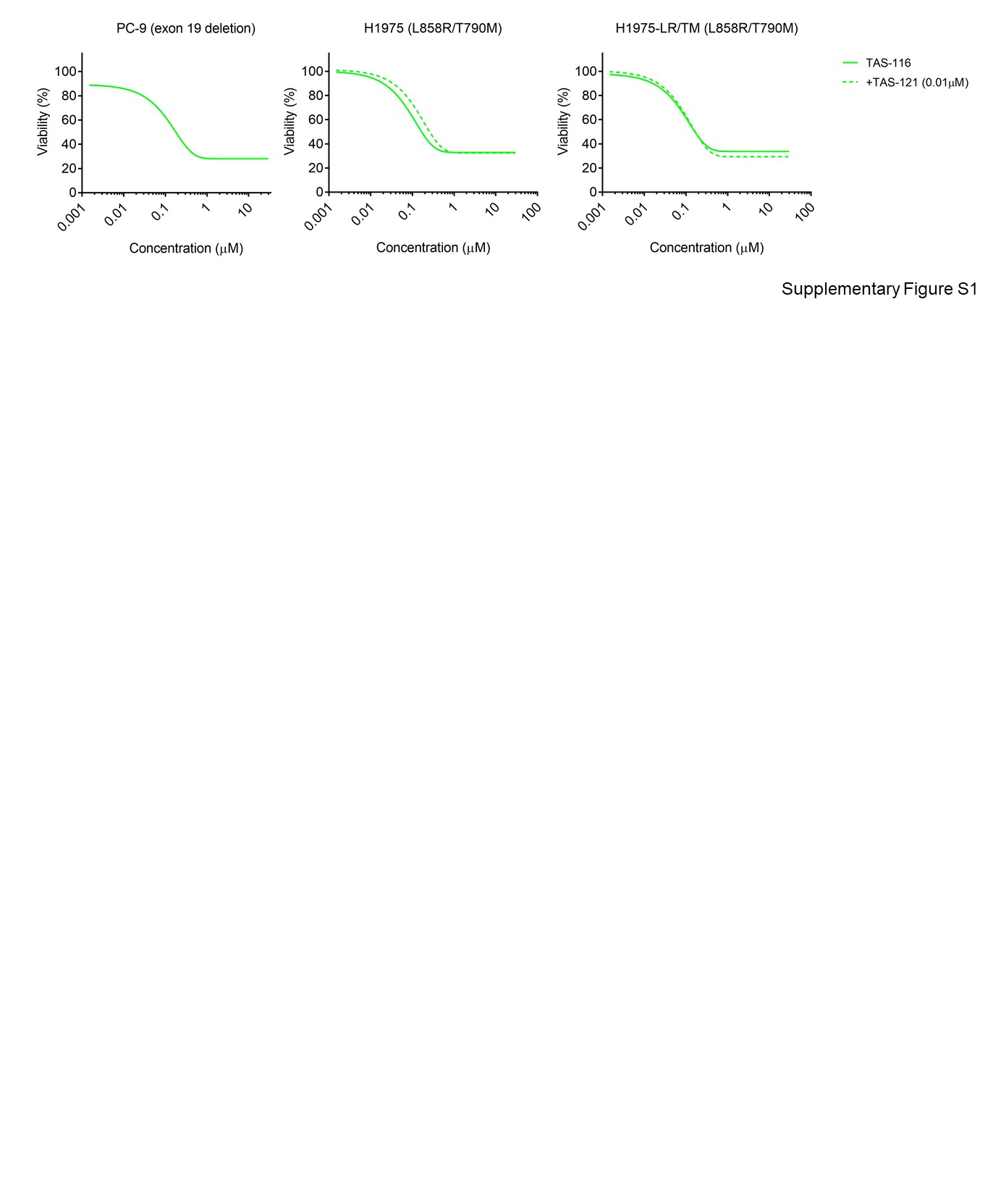
Cells were plated in 96-well plates at a density of 2 × 10^3^ cells/well, and cell proliferation was evaluated with a WST-1 assay after 72 hours of treatment. Experiments were performed independently in triplicate.

**Supplementary Table S1.** Lists of 104 genes examined by NCC Oncopanel v3 test.

| **Mutation and copy number alterations for all exons** | | | | | **Fusion** |
| --- | --- | --- | --- | --- | --- |
| *ABL1* | *CREBBP* | *IL7R* | *NTRK1* | *SMAD4* | *ALK* |
| *ACTN4* | *CTNNB1* | *JAK1* | *NT5C2* | *SMARCA4* | *AKT3* |
| *AKT1* | *CUL3* | *JAK2* | *PALB2* | *SMO* | *AXL* |
| *AKT2* | *DDR2* | *JAK3* | *PBRM1* | *STAT3* | *BRAF* |
| *AKT3* | *EGFR* | *KEAP1* | *PDGFRA* | *STK11* | *EGFR* |
| *ALK* | *ENO1* | *KIT* | *PDGFRB* | *TP53* | *ERBB4* |
| *APC* | *EP300* | *KRAS* | *PIK3CA* | *TSC1* | *FGFR1* |
| *ARID1A* | *ERBB2* | *MAP2K1* | *PIK3R1* | *VHL* | *FGFR2* |
| *ARID2* | *ERBB3* | *MAP2K4* | *PIK3R2* |  | *FGFR3* |
| *ATM* | *ERBB4* | *MAP3K1* | *PRKCI* |  | *NOTCH1* |
| *AXIN1* | *ESR1* | *MAP3K4* | *PTCH1* |  | *NRG1* |
| *AXL* | *EZH2* | *MDM2* | *PTEN* |  | *NTRK1* |
| *BAP1* | *FBXW7* | *MDM4* | *RAC1* |  | *PDGFRA* |
| *BARD1* | *FGFR1* | *MET* | *RAC2* |  | *RAF1* |
| *BCL2L11* | *FGFR2* | *MTOR* | *RAD51C* |  | *RET* |
| *BRAF* | *FGFR3* | *MYC* | *RAF1* |  | *ROS1* |
| *BRCA1* | *FGFR4* | *MYCN* | *RB1* |  |  |
| *BRCA2* | *FLT3* | *NF1* | *RET* |  |  |
| *CCND1* | *GNAS* | *NFE2L2* | *RHOA* |  |  |
| *CD274* | *HRAS* | *NOTCH1* | *ROCK1* |  |  |
| *CDK4* | *IDH1* | *NOTCH2* | *ROCK2* |  |  |
| *CDKN2A* | *IDH2* | *NOTCH3* | *ROS1* |  |  |
| *CHEK2* | *IGF1R* | *NRAS* | *SETBP1* |  |  |
| *CRKL* | *IGF2* | *NRG1* | *SETD2* |  |  |

**Supplementary Table S2.** *EGFR* mutational statuses of cell lines.

| Cell line | Genetic alterations |
| --- | --- |
| HCC827 | *EGFR* E746_A750del |
| PC-9 | *EGFR* E746_A750del |
| PC9-COR | Wild-type *EGFR* amplification |
| H1975 | *EGFR* L858R/T790M |
| H1975-LR/TM | *EGFR* L858R/T790M + *EGFR* L858R/T790M overexpression |

**Supplementary Table S3.** Summary of antibodies for western blotting.

| Molecule | Clone | Host | Company |
| --- | --- | --- | --- |
| Human EGFR | D38B1 | Rabbit | Cell Signaling Technology |
| Human pEGFR | D7A5 | Rabbit | Cell Signaling Technology |
| Human ex 19 deleted EGFR | D6B6 | Rabbit | Cell Signaling Technology |
| Human AKT | Polyclonal | Rabbit | Cell Signaling Technology |
| Human pAKT (Ser473) | D9E | Rabbit | Cell Signaling Technology |
| Human ERK1/2 | 137F5 | Rabbit | Cell Signaling Technology |
| Human pERK1/2 | D13.14.4E | Rabbit | Cell Signaling Technology |
| Human GAPDH | D16H11 | Rabbit | Cell Signaling Technology |

**Supplementary Table S4.** Summary of genetic alterations.

| Mutation | | Fusion | Copy number variation |
| --- | --- | --- | --- |
| *EGFR* L858R | Not detected | | *NOTCH2* amplification |
| *EGFR* T790M |  | | *RAC1* amplification |
| *TP53* L194fs |  | | *EGFR* amplification |
|  |  | | *CDKN2A* homozygous deletion |
